# Supplementary material for: How Electric Field Remodels the Nanofibril Structure of Chitosan Hydrogels: the Role of Dewetting During Electro-Assembly
Source: bioRxiv. 2025 Jul 8:2025.04.25.650622. Preprint. [Version 4] doi: 10.1101/2025.04.25.650622 (PMC12265699; doi:10.1101/2025.04.25.650622)
Supplement: Supplement 1 [file media-1.pdf]

Supporting Information: How Electric Field Remodels the Nanofibril Structure of Chitosan Hydrogels: the Role of Dewetting During Electro-Assembly

Aarion Romany,[†] Gregory F. Payne,[‡] and Jana Shen[*,†]

† *Department of Pharmaceutical Sciences, University of Maryland School of Pharmacy, Baltimore, MD 21201*

‡ *Institute for Bioscience and Biotechnology Research, University of Maryland, College Park, MD 20742*

E-mail: jana.shen@rx.umaryland.edu

List of Tables

List of Figures

S1 Time series of interchain hydrogen bonds and solvent exposure of the chitosan nanofibril with an e-field S-3

S2 Time series of interchain hydrogen bonds and solvent exposure of the chitosan nanofibril without an e-field S-4

S3 Time series of the intrachain hydrogen bonds with and without an e-fied . . S-5

S4 Time series of the change in the nanofibril volume with and without an e-fieldS-6

S5 Time series of sheet stacking distance (thickness) with and without e-field . S-7

S6 Distribution of sheet growth distance with and without e-field S-8

Supplemental figures

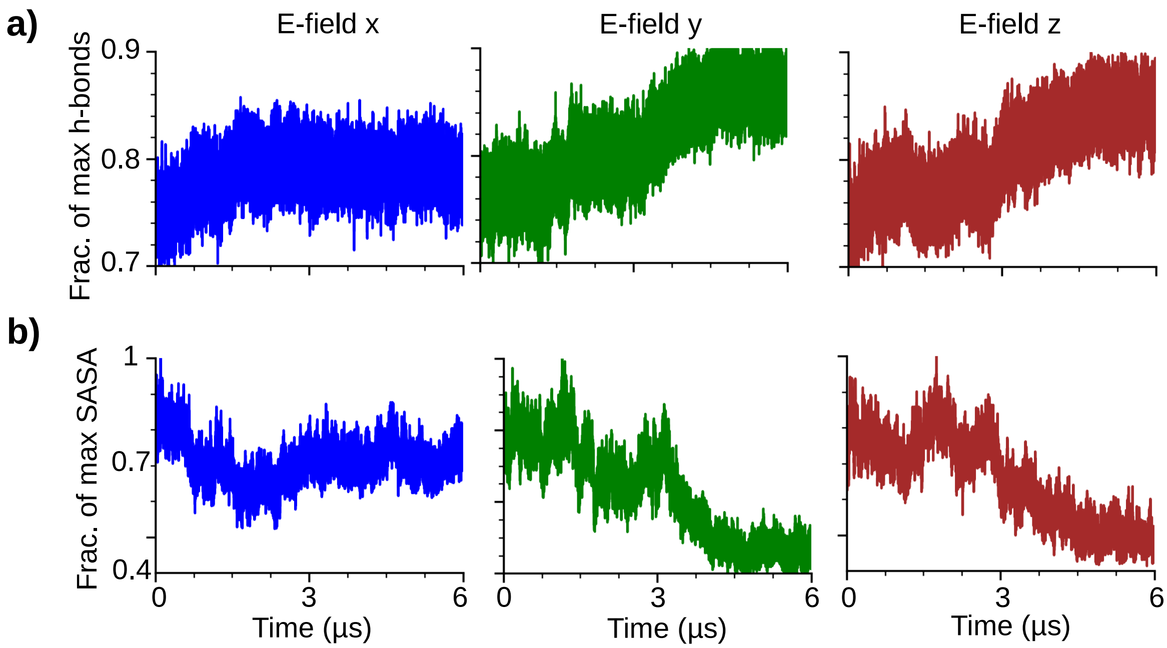

Figure S1: Time series of **(a)** the fraction of max interchain hydrogen bonds and **(b)** solvent accessible surface area (SASA) of the chitosan nanofibril under the electric field in x (left), y (middle), and z (right) direction. A hydrogen bond is considered present if the heavy-atom donor-acceptor distance is below 3.5 Å and the donor-hydrogen-acceptor angle greater than 135°. Note, all 24 chains were used. Fraction is calculated relative to the fibril after energy minimization.

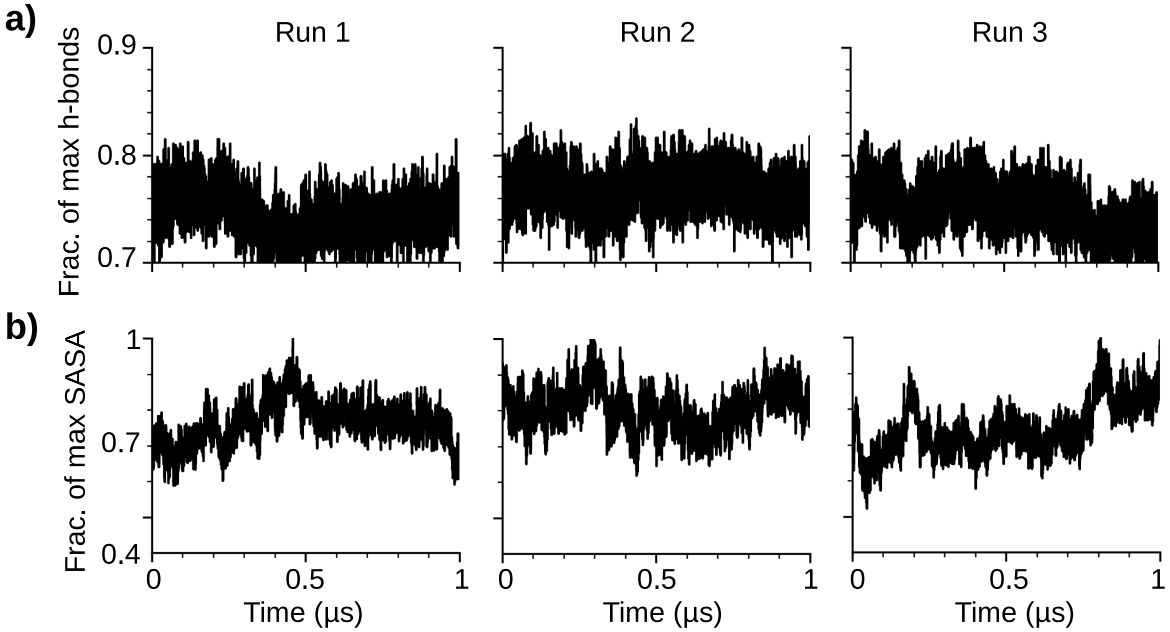

Figure S2: Time series of **(a)** the fraction of max interchain hydrogen bonds and **(b)** the fraction of max SASA in three independent simulations without e-field applied.

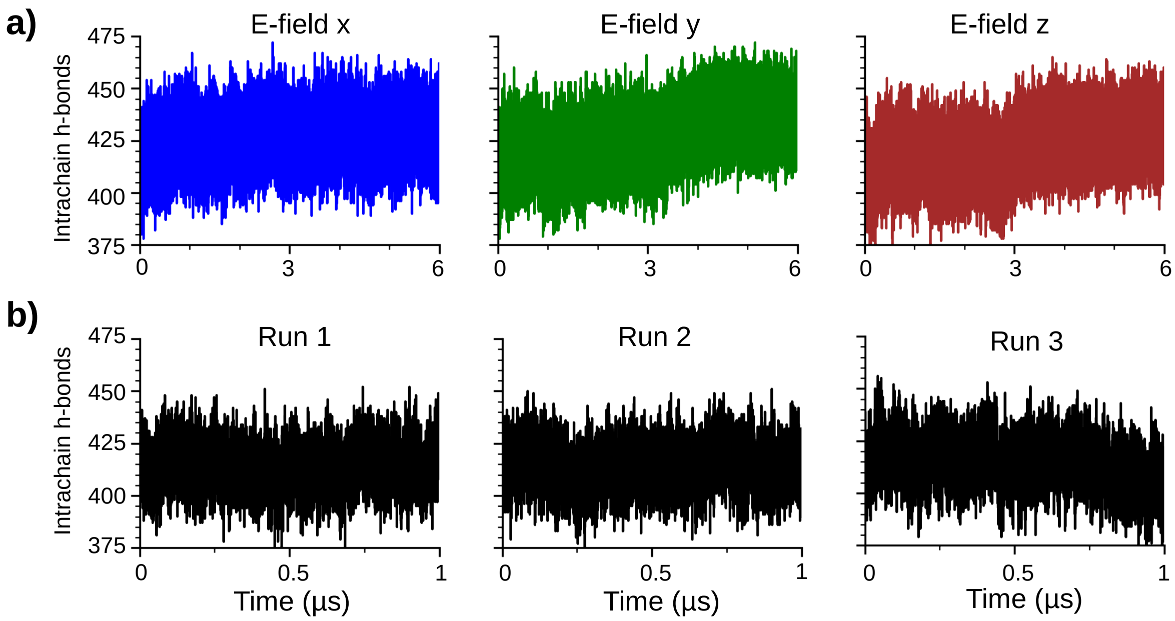

Figure S3: Time series of the total number of intrachain hydrogen bonds in the chitosan nanofibril **(a)** in the simulations with the e-field and **(b)** without an e-field.

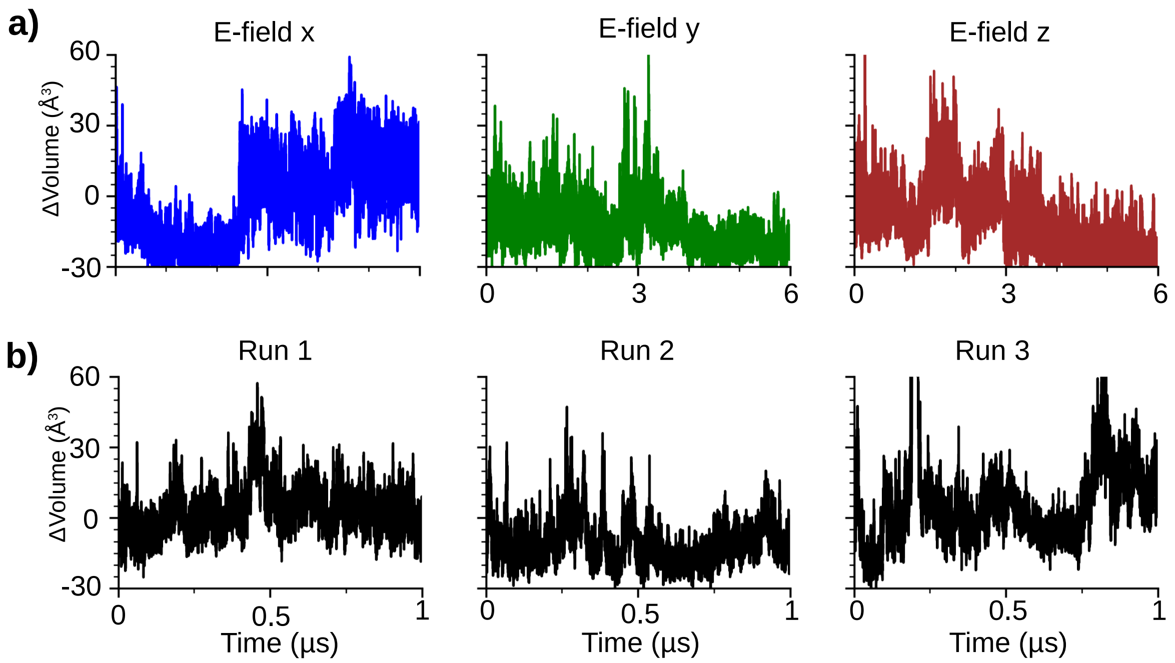

Figure S4: Time series of the change in the nanofibril volume **(a)** with and **(b)** without an e-field applied. The change is calculated relative to the volume of the chitosan nanofibril at the beginning of the production run.

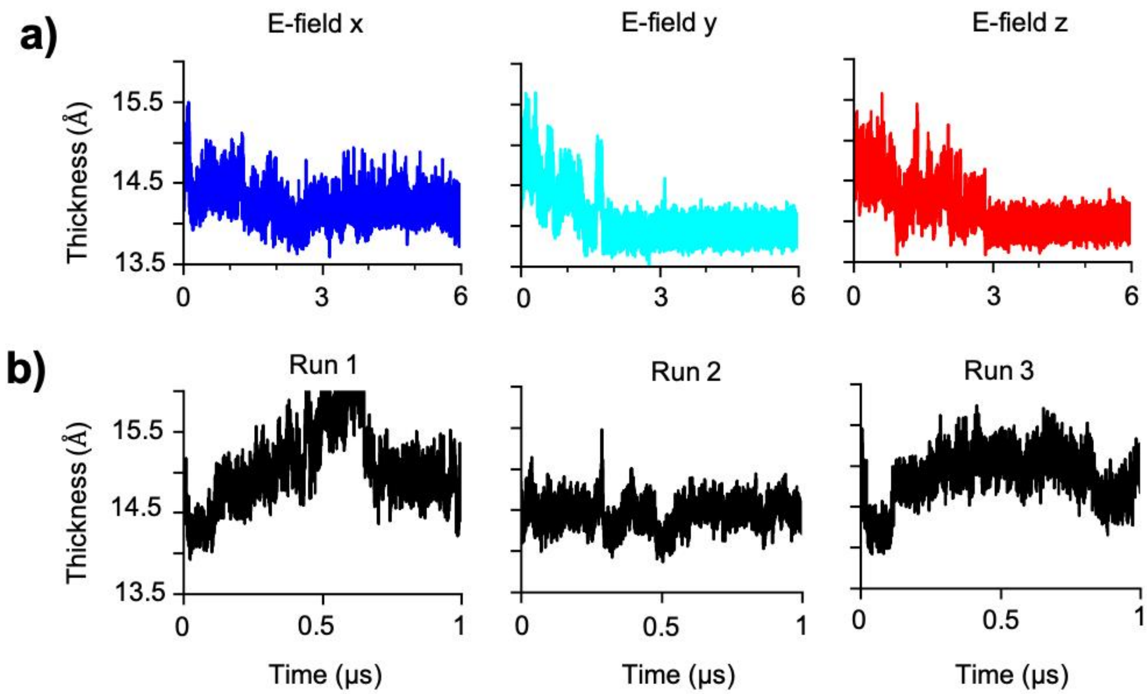

Figure S5: Time series of the change in the nanofibril Thickness **(a)** with and **(b)** without an e-field applied. The thickness is calculated as the distance between center chains in sheet 1 and sheet 4.

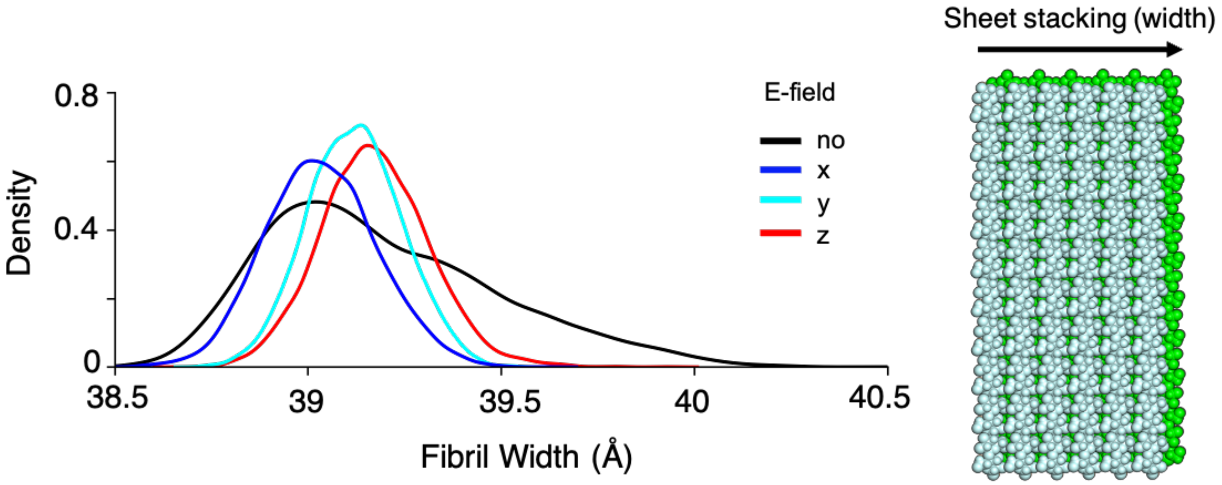

Figure S6: Probability distribution (left) of the distance along the sheet growth direction with and without an e-field applied. It is calculated as the center of mass distances between the two end chains of the inner sheets. Chitosan nanofibril (right) showing the distance illustrating the distance measured.
